# Supplementary material for: Excitonic linewidth and coherence lifetime in monolayer transition metal dichalcogenides
Source: Nat Commun. 2016 Nov 7;7:13279. doi: 10.1038/ncomms13279 (PMC5103057; doi:10.1038/ncomms13279)
Supplement: Supplementary Information — Supplementary Figure 1, Supplementary Note 1 and Supplementary Reference [file ncomms13279-s1.pdf]

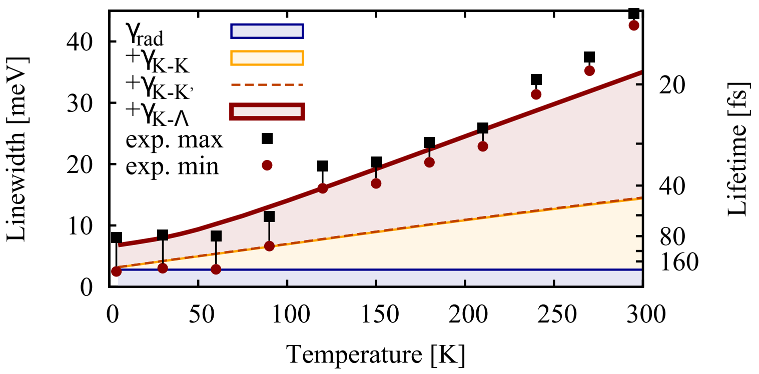

Supplementary Figure 1: **Excitonic linewidth and lifetime in WSe_2.** Temperature dependence of the linewidth and lifetime of the A exciton in WSe_2 as a function of temperature. The red and black points correspond to the minimum and maximum limits of the homogeneous linewidths extracted from experiment, respectively (see Methods section). The thick red line shows the total linewidth consisting of the single contributions from the radiative decay γ_{rad} (blue) and non-radiative decay including intravalley exciton-phonon coupling $\gamma^{KK}_{\text{non-rad}}$ (orange) as well as intervalley coupling $\gamma^{K\Lambda}_{\text{non-rad}}$ and $\gamma^{KK'}_{\text{non-rad}}$ (dashed orange). Note that the latter contribution is very small.

Supplementary Note 1 - Homogeneous linewidth in WSe_2

In Fig. S1 we show the computed and measured excitonic linewidth for monolayer WSe_2. Fitting our results to the form $\gamma = \gamma_0 + c_1 T + \frac{c_2}{e^{\frac{\Omega}{kT}} - 1}$, we obtain γ_0 =6.4 meV (consisting of 2.8 meV due to radiative decay), c_1 =56 μ eV K^{-1} , c_2 =9.4 meV and Ω =15 meV. According to our calculations, the dark $K - \Lambda$ exciton states are energetically located approximately 70 meV below the bright states in $K - K$ valley. The strong impact of scattering into these states even at very low temperatures again illustrates the clear distinction between molybdenum and tungsten based TMDs. Comparing the linewidth at low temperatures with those in WS_2, cf. Fig. 3 (a) in the main manuscript, we find an even greater homogeneous

broadening due to the emission of Λ phonons. This effect originates from the lower phonon energies in WSe_2 [1].

[1] Jin, Z., Li, X., Mullen, J. T. & Kim, K. W. Intrinsic transport properties of electrons and holes in monolayer transition-metal dichalcogenides. *Phys. Rev. B* **90**, 045422 (2014).
